# The Role and Mechanisms of miRNAs on Ovarian Granulosa Cells: A Literature Review

**DOI:** 10.3390/genes17020121

**Published:** 2026-01-24

**Authors:** Siyu Chen, Jiawei Lu, Yuqian Si, Lei Chen, Ye Zhao, Lili Niu, Yan Wang, Xiaofeng Zhou, Linyuan Shen, Ya Tan, Li Zhu, Mailin Gan

**Affiliations:** 1 Farm Animal Germplasm Resources and Biotech Breeding Key Laboratory of Sichuan Province, Sichuan Agricultural University, Chengdu 611130, China; 2 State Key Laboratory of Swine and Poultry Breeding Industry, Sichuan Agricultural University, Chengdu 611130, China; 3 Key Laboratory of Livestock and Poultry Multi-Omics, Ministry of Agriculture and Rural Affairs, College of Animal and Technology, Sichuan Agricultural University, Chengdu 611130, China; 4 Institute of Animal Husbandry and Veterinary, Guizhou Academy of Agricultural Science, Guiyang 550005, China

**Keywords:** miRNAs, follicular atresia, granulosa cells, oxidative stress, research progress

## Abstract

**Background**: Ovarian granulosa cells (GCs) play a pivotal role in folliculogenesis, and their dysfunction is central to disorders such as polycystic ovary syndrome (PCOS) and premature ovarian failure (POF). MicroRNAs (miRNAs) have emerged as crucial post-transcriptional regulators of GC homeostasis. **Method**: This review synthesizes current evidence by systematically analyzing relevant studies, integrating data from in vitro GC models, animal experiments, human cell lines, and clinical samples to elucidate the specific mechanisms by which miRNAs regulate GCs. **Results**: miRNAs precisely modulate GC proliferation, apoptosis, steroidogenesis, and oxidative stress responses by targeting key signaling pathways (e.g., PI3K/AKT/mTOR, TGF-β/SMAD) and functional genes (e.g., *TP53*, *CYP19A1*). Exosomal miRNAs serve as vital mediators of communication within the follicular microenvironment. To date, nearly 200 miRNAs have been associated with PCOS. **Conclusions**: miRNAs constitute a decisive regulatory network governing GC fate, offering promising therapeutic targets for PCOS and POF. However, significant challenges remain, primarily miRNA pleiotropy and the lack of follicle-specific delivery systems. Future clinical translation requires rigorous validation in human-relevant models.

## 1. Introduction

Ovarian granulosa cells (GCs) are the essential somatic microenvironment for oocytes, governing folliculogenesis through metabolic support, steroid hormone synthesis, and precise regulation of proliferation and apoptosis. Dysregulation of GC homeostasis is a hallmark of polycystic ovary syndrome (PCOS) and premature ovarian failure (POF), which are disorders affecting millions of women worldwide [1]. The functional integrity of GCs determines follicular fate, with external stressors further compromising their function and highlighting their central importance in female reproductive health.

MicroRNAs (miRNAs), approximately 22-nucleotide non-coding RNAs, have emerged as dominant post-transcriptional regulators across biological systems. Recent genomic studies have expanded their functional repertoires dramatically: systematic re-analysis of 14,035 small-RNA-seq libraries uncovered >6000 newly annotated candidate loci, approximately doubling the searchable repertoire for granulosa-cell studies and revealing ovary-enriched clusters that had escaped canonical annotations [2]. Given this pivotal role in gene regulation and intricate link to reproductive pathologies, miRNAs have justifiably become a major research focus [3].

In the ovarian context, miRNAs serve dual functions: they regulate gene expression cell autonomously while also mediating intercellular communication via extracellular vesicles, particularly exosomes. These vesicles enable trafficking of functional miRNAs between cells within the follicular niche, with exosomal miRNAs derived from sources such as stem cells directly modulating GC gene expression and steroidogenic function [4]. This convergence of GC biology and miRNA mechanisms underscores a vital research frontier.

The clinical urgency is underscored by demographic trends: ovarian function declines with age, a process significantly accelerated by modern lifestyle factors such as circadian disruption and hypercaloric intake, which correlate with altered follicular miRNA profiles in young women. Accumulating evidence indicates that clinical control of follicular atresia, initiated by GC apoptosis, is crucial to alleviate diseases such as PCOS and POF. During gonadotropin-induced follicular development, GC mitochondria generate ATP via oxidative phosphorylation, while reactive oxygen species (ROS) production increases dramatically. Excessive ROS accumulation induces oxidative damage, mitochondrial DNA mutations, and lipid peroxidation, ultimately compromising GC-oocyte communication and oocyte quality [5].

This review systematically synthesizes current evidence on miRNA regulation of GC proliferation, apoptosis, steroidogenesis, and oxidative stress responses. By integrating data from in vitro GC cultures, animal models (mouse, rat, goat, sheep, pig, chicken), human granulosa-like tumor cell lines, and clinical samples—as exemplified by studies that map miRNA-mRNA interactomes in specific models [6]—we elucidate specific mechanisms by which miRNAs target key signaling pathways and functional genes. We emphasize that nearly 200 miRNAs are now associated with PCOS, yet the field faces significant translational challenges, primarily miRNA pleiotropy and the absence of follicle-specific delivery systems [3]. Most evidence derives from in vitro cultures or animal models, highlighting the critical need for validation in human-relevant systems such as organoids before miRNA-based strategies can be considered viable clinical tools for managing ovarian dysfunction. Deciphering the specific miRNA networks that control GC proliferation, apoptosis, and differentiation is therefore a research frontier of both fundamental and translational importance.

## 2. miRNAs Regulating Proliferation and Apoptosis of Ovarian Granulosa Cells

Post-transcriptional control by miRNAs constitutes a decisive layer governing granulosa-cell fate. The latest release of miRNA databases (e.g., mirDB 4.0) incorporates > 6000 newly annotated human miRNA loci—many enriched in ovarian clusters—providing an expanded repertoire for granulosa-cell-specific target prediction and functional annotation. Recent omics-level datasets now offer a systems-level view of these interactions. By integrating 12 shared miRNAs with matched mRNA profiles across two GC sub-populations, Yousuf et al. constructed a high-confidence miRNA–mRNA interactome that positions miR-129, miR-30a-5p, and miR-146a-5p as master regulators of proliferation vs. apoptosis hubs in human granulosa cells [6]. This extensive miRNA network directly targets key genes controlling cell survival, with major implications for follicular atresia and PCOS development [1,7].

### 2.1. miRNAs Targeting Core Proliferation and Apoptosis Genes

MiRNAs directly target key genes controlling cell survival, with major implications for follicular atresia and PCOS development. The tumor suppressor *TP53* is a critical regulator of GC apoptosis, and several miRNAs modulate its expression. It has been demonstrated that miR-644-5p, delivered via bone mesenchymal stem cell (BMSC)-derived exosomes, regulates p53 to inhibit ovarian granulosa cell apoptosis, showing therapeutic potential for POF [8]. In contrast, Xie et al. found that miR-4110 increases Sp1 expression by suppressing Smad2, leading to p53 upregulation, increased Bax levels, and ultimately GC apoptosis [9]. Interestingly, p53 can also regulate miRNA expression: Liang et al. reported that p53 binds to the proximal promoter region of the GABAA receptor ε subunit of the miR-224 host gene, regulating miR-224 and SMAD4 expression, consequently affecting GC proliferation and estradiol release [10]. Additionally, Tao et al. showed that p53 inhibits miR-27a expression by targeting its promoter, resulting in upregulation of *NFAT5* and promotion of mouse granulosa cell proliferation [11].

Potassium voltage-gated channel subfamily A member 5 (*KCNA5*) represents another important target. In PCOS, miR-3188 has been identified as a key miRNA that inhibits apoptosis and promotes GC proliferation through targeting *KCNA5*, with downstream upregulation of Bcl-2 and downregulation of Bax and Caspase-3 [12]. Similarly, miR-3940-5p was shown to target *KCNA5*, thereby promoting GC proliferation in PCOS [13].

Mitochondrial fusion proteins Mfn1 and Mfn2 are essential for female fertility. Hou et al. showed that Mfn1 deficiency leads to follicular development arrest and mitochondrial dysfunction in oocytes [14]. Wang et al. linked low Mfn2 expression in GCs with age and worse ART outcomes by impairing mitochondrial function and promoting apoptosis [15]. Shi et al. reported that miR-214-3p directly regulates Mfn2, inhibiting GC proliferation and estradiol synthesis [16]. These findings highlight mitochondrial fusion proteins as critical, though understudied, miRNA targets in GC biology.

Additional key functional genes are regulated by miRNAs. miR-107 suppresses porcine GC proliferation and estradiol synthesis while promoting apoptosis by targeting *PTGS2* [17]. Wang et al. demonstrated that miR-17-5p transcriptionally activates KPNA2 expression via a non-canonical RNA activation mechanism, counteracting high-glucose-induced oxidative stress and apoptosis in sheep granulosa cells [18]. The insulin-like growth factor pathway is also heavily targeted: The IGF-1 pathway is heavily targeted by miRNAs in various contexts. For instance, exposure to bisphenol A can dysregulate the IGF-1/miR-27b-3p axis [19], while in PCOS, mechanisms such as lncRNA HCP5 competitively binding miR-27a-3p [20] or miR-130a directly reducing IGF-1 expression have been reported [21]; Geng et al. demonstrated that miR-99a regulates proliferation and apoptosis of human GCs by targeting *IGF1R* [22]; Wu et al. found that miR-323-3p regulates steroidogenesis and apoptosis in KGN cells by targeting IGF-1 [23]; Zhong et al. showed that inhibition of miR-19b promotes GC proliferation by targeting IGF-1 in PCOS [24]; and Yang et al. revealed that miR-451a targets the ATF2 signaling pathway, modulating GC proliferation and apoptosis in a rat PCOS model [25]. Key miRNAs and their target genes discussed above are summarized in Table 1.

**Table 1:** Key miRNAs regulating core proliferation and apoptosis genes in granulosa cells.

Target Gene/Pathway	Regulatory miRNA(s)	Effect on GCs	Associated Context	Reference
*TP53*/p53 axis	miR-644-5p (↑)	Inhibits apoptosis	POF therapy	[8]
	miR-4110 (↓)	Promotes apoptosis via Sp1/p53		[9]
	p53 → miR-27a (↓)	Promotes proliferation via NFAT5		[11]
*KCNA5*	miR-3188, miR-3940-5p (↑)	Promote proliferation, inhibit apoptosis	PCOS	[12,13]
Mitofusins (Mfn1/2)	miR-214-3p (↑)	Inhibits proliferation & E2 synthesis	Porcine model	[16]
IGF-1/*IGF1R* axis	miR-27a-3p, -130a, -99a, -323-3p, -19b (↓)	Mostly inhibit proliferation/promote apoptosis	PCOS models	[19,20,21,22,23,24]
*PTGS2*	miR-107 (↑)	Suppresses proliferation, promotes apoptosis	Porcine GCs	[17]
*KPNA2*	miR-17-5p (↑, via RNAa)	Inhibits HG-induced apoptosis	Sheep GCs	[18]
ATF2 pathway	miR-451a (↑)	Modulates proliferation/apoptosis	Rat PCOS model	[25]

Note: Arrows (↑ and ↓) denote upregulation and downregulation of miRNA expression, respectively, under the specified conditions.

### 2.2. miRNAs Regulating Signaling Pathway Activity

MiRNAs precisely modulate core signaling cascades, with pathway-level integration determining GC fate decisions.

#### 2.2.1. PI3K/AKT/mTOR Signaling Pathway

**Figure 1:**
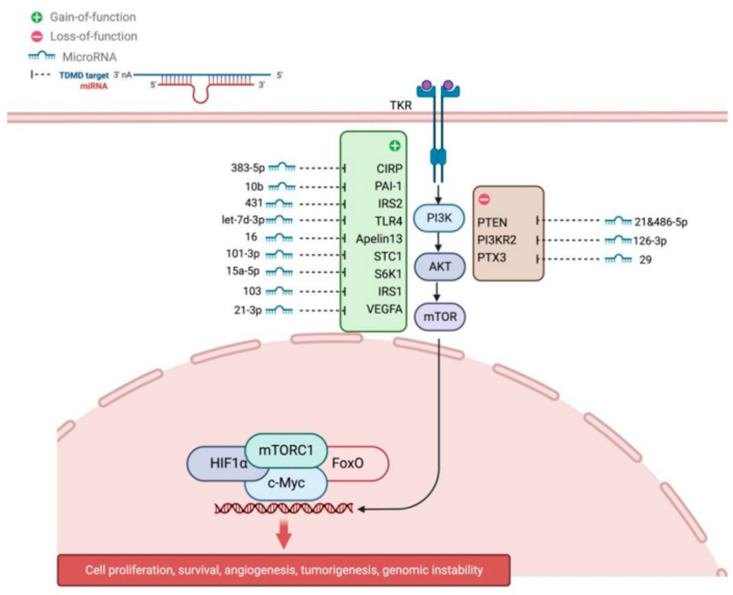
miRNAs regulating the PI3K/AKT/mTOR signaling pathway. This schematic illustrates complex post-transcriptional regulation of the central PI3K/AKT/mTOR cascade by multiple miRNAs. Inhibitory miRNAs (red lines) suppress pathway components: let-7d-3p targets *TLR4*; miR-431 targets *IRS2*; miR-10b targets *PAI-1*; miR-101-3p targets *STC1*; and *PTEN* acts as a natural brake by converting PIP3 to PIP2. Modulatory miRNAs (black arrows) target regulatory nodes: miR-126-3p targets *PIK3R2*; miR-29 targets *PTX3*; miR-21-3p targets *VEGFA*; miR-103 modulates *mTOR*; miR-15a-5p targets *IRS1*; miR-383-5p targets *CIRP*; and miR-486-5p targets *APPL1*. Upon activation, PI3K phosphory lates PIP2 to PIP3, leading to AKT phosphorylation and mTORC1 activation. Downstream effectors regulate proliferation, survival, and metabolism.

The PI3K/AKT/mTOR axis is central to GC proliferation and follicle dominance. In PCOS, miR-let-7d-3p inhibits the PI3K/Akt pathway and GC proliferation by targeting *TLR4* [23]. Guo et al. demonstrated that miR-10b promotes apoptosis by targeting *PAI-1* to regulate the PI3K/AKT pathway [24]. An et al. showed that miR-101-3p inhibits PI3K/AKT/mTOR by targeting *STC1* in goat ovarian development [26]. High levels of miR-15a-5p correlate with poor ovarian response, likely via inhibiting granulosa cell proliferation through this pathway [27]. Yang et al. reported that miR-431 inhibits GC proliferation by targeting *IRS2* and suppressing PI3K/AKT signaling [28]. Wang et al. found that miR-29 regulates goat GC function by targeting *PTX3*, activating both PI3K/AKT/mTOR and Erk1/2 pathways [29]. Han et al. demonstrated that miR-21 in oocyte secretory factors inhibits PI3K/Akt signaling and promotes GC apoptosis [30]. Recent work in sheep showed that miR-134-3p suppresses proliferation and induces apoptosis by targeting *INHBA* and dampening the TGF-β/PI3K/AKT cascade [31]. Clinically, Han et al. discovered that *PD-L1* upregulation in PCOS suppresses GC apoptosis by activating PI3K/AKT, highlighting a potential immune-endocrine axis [32]. The miRNA-mediated regulation of PI3K/AKT/mTOR signaling is depicted in Figure 1.

Beyond the well-characterized miRNA regulators, emerging evidence from high-throughput screening has identified additional miRNA-target interactions that modulate PI3K/AKT/mTOR signaling, though their functional roles in granulosa cells remain to be fully validated. These include miR-126-3p targeting *PIK3R2* (the regulatory subunit of PI3K), miR-103 directly regulating *mTOR* expression, and miR-21-3p controlling *VEGFA*-mediated angiogenic signals. A study in endometrial cancer cells demonstrated that miR-126-3p directly binds the 3′UTR of *PIK3R2*, reducing PI3K activity and downstream AKT phosphorylation [33]. Similarly, miR-103 has been shown to suppress *mTOR* expression in hepatocellular carcinoma, influencing cell metabolism and proliferation [34]. While these specific interactions have not yet been confirmed in ovarian granulosa cells, the conserved nature of miRNA targeting suggests they may represent novel regulatory nodes in follicular development. Additionally, the cold-inducible RNA-binding protein (CIRP) has been identified as a target of miR-383-5p in stress responses [35], and the tumor suppressor *PTEN* is co-targeted by miR-21 and miR-486-5p within the PI3K/AKT/mTOR network [36,37], offering potential new avenues for investigating granulosa cell metabolism and survival.

#### 2.2.2. JAK/STAT and Hippo Signaling Pathways

The JAK/STAT pathway is essential for cytokine signaling in GCs. Frost et al. reported conserved roles in human follicle development [38]. Hall et al. confirmed PI3K/AKT and JAK/STAT signaling as conserved biomarkers for ovarian development [39]. Yang et al. showed that IL-6 promotes FSH-induced VEGF expression through JAK/STAT3 in bovine GCs [40]. Ndiaye et al. identified novel JAK3-interacting proteins in GCs, including *LEPROTL1*, *INHBA*, and *CDKN1B* [41]. The highly expressed miR-199a-5p in PCOS patients inhibits *WT1*-mediated JAK/STAT3 activation, and its own inhibition has been shown to improve PCOS pathology [42].

**Figure 2:**
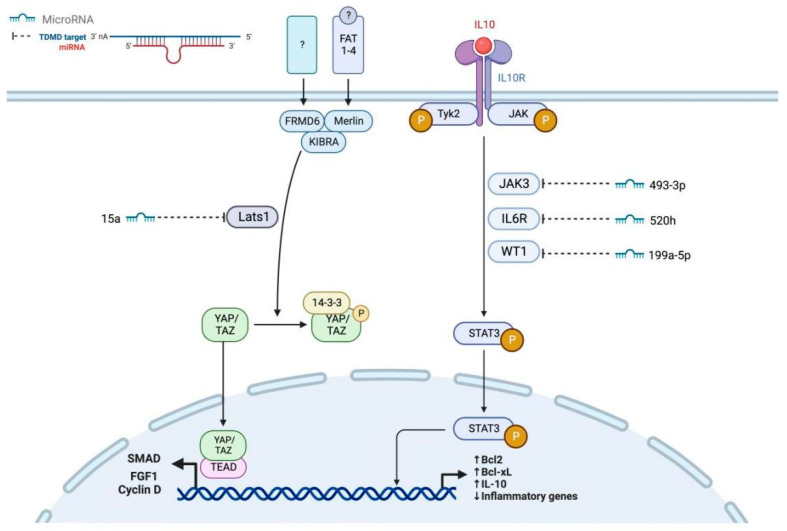
miRNAs regulating JAK/STAT3 and Hippo signaling pathways. This schematic depicts the crosstalk between two critical signaling axes. Hippo pathway (**Left**): Upstream proteins Merlin, KIBRA, FAT, and FRMD6Y phosphorylate and activate Lats1 kinase. Lats1 subsequently phosphorylates *YAP*/*TAZ*, sequestering them in the cytoplasm via 14-3-3 binding and preventing *TEAD*-mediated transcription of downstream genes, including *Bcl2*, *Bcl-xL*, *CyclinD*, and *FGF1*. The miRNA miR-15a directly targets *Lats1* (red inhibition line), derepressing *YAP*/*TAZ* nuclear translocation. JAK/STAT3 Pathway (**Right**): Ligands bind to cytokine receptors *IL10R* and *IL6R*, activating *Tyk2* and *JAK3* kinases. Phosphorylated STAT3 dimerizes, translocates to the nucleus, and drives transcription of target genes, including *Bcl2*, *Bcl-xL*, *CyclinD*, *FGF1*, and inflammatory mediators. The miRNAs miR-199a-5p, miR-520h, and miR-493-3p interface with pathway components by targeting *WT1*, *IL6R*, and *IL10R*, respectively (note: connection directionality is not specified in the diagram). Pathway Crosstalk: Both pathways converge on the regulation of proliferation and apoptosis-related genes (*Bcl2*, *Bcl-xL*, *CyclinD*, *FGF1*). The Hippo pathway primarily controls developmental signals, while *JAK*/*STAT3* mediates inflammatory and survival responses.

The Hippo pathway is emerging as a critical regulator. Ai et al. determined that miR-15a targets *Lats1* to inhibit GC proliferation and induce senescence. Recent studies expand Hippo crosstalk: Wang et al. demonstrated that *RSPO2* coordinates with *GDF9*:*BMP15* heterodimers to promote GC development via Wnt/β-catenin signaling, which extensively crosstalks with Hippo [43]. Shan et al. identified miR-184 as a transcriptional activator that acts as a transcriptional enhancer that indirectly induces *SMAD3* expression by targeting an inhibitory factor, potentially interfacing with Hippo through TGF-β crosstalk [44]. The cytokine sensitivity of granulosa cells is further fine-tuned by miRNAs targeting receptor components of the JAK/STAT pathway. Recent transcriptomic analyses have identified miR-520h as a post-transcriptional regulator of *IL6R* expression, modulating granulosa cell responsiveness to IL-6-mediated signals [45]. Similarly, miR-493-3p has been implicated in controlling *MIF* (macrophage migration inhibitory factor) expression, thereby influencing macrophage infiltration and inflammatory responses [46]. While these specific receptor-targeting mechanisms have been characterized primarily in immune cells, their presence in ovarian granulosa cell transcriptome datasets suggests they may play important roles in regulating the follicular immune microenvironment during folliculogenesis and atresia. Validation of these interactions in granulosa cell-specific models represents an important area for future investigation. Taken together, these recent studies highlight that the Hippo pathway does not act in isolation; rather, through extensive crosstalk with other key pathways such as Wnt/β-catenin and TGF-β, it forms an integral part of the complex network determining granulosa cell fate. The miRNAs regulating JAK/STAT3 and Hippo signaling pathways are illustrated in Figure 2. 

#### 2.2.3. MAPK Signaling Pathway

**Figure 3:**
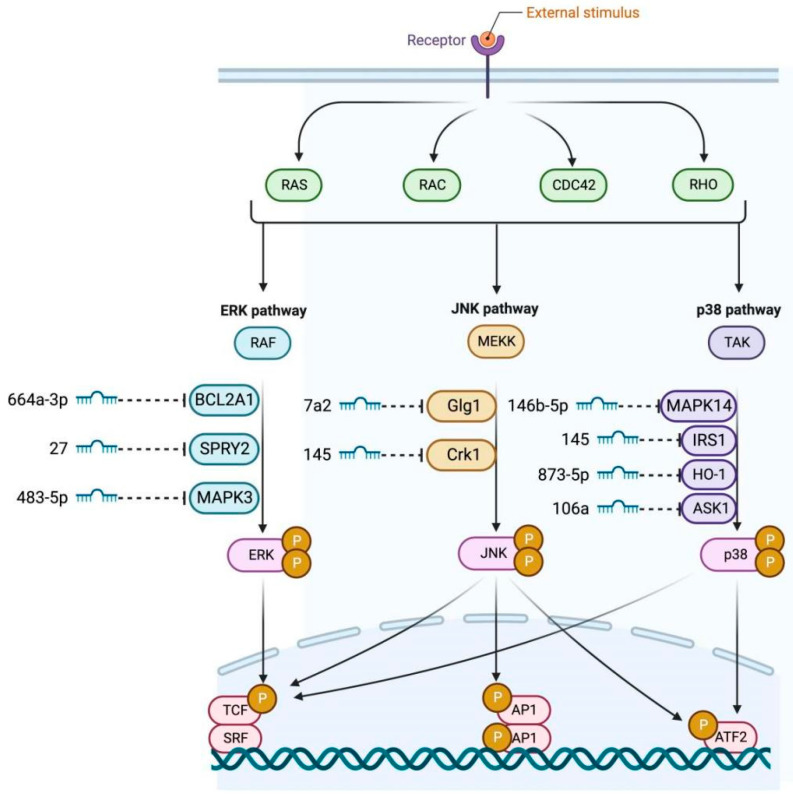
miRNAs regulating the MAPK signaling pathway. This schematic depicts an integrated network where external stimuli activate receptors to stimulate small GTPases (RAS, RAC, CDC42, RHO), initiating three convergent MAPK cascades. The ERK branch signals through RAF, the JNK branch through MEKK, and the p38 branch through TAK/ASK1, ultimately activating ERK, JNK, and p38 kinases, respectively. Multiple miRNAs exert inhibitory control: miR-664a-3p targets *BCL2A1*; miR-7a-2-3p targets *Glg1*; miR-146b-5p targets *MAPK14*; miR-27a targets *SPRY2*; miR-145 targets *IRS1* and *Crk1*; miR-873-5p targets HO-1; miR-483-5p targets *MAPK3*; and miR-106a targets ASK1. These cascades coalesce on common transcription factors (TCF, AP1, SRF, ATF2) that orchestrate cell proliferation, differentiation, and stress responses.

The MAPK pathway is a major signal transducer in GCs. miR-664a-3p inhibits KGN cell proliferation and promotes apoptosis by targeting *BCL2A1* and consequently blocking MAPK/ERK signaling [47]. Liu et al. found that miR-146b-5p overexpression attenuates premature ovarian failure in mice by inhibiting the Dab2ip/Ask1/p38MAPK pathway [7]. Hong et al. showed that miR-106a increases GC viability by inhibiting *ASK1* mRNA and p38MAPK phosphorylation [48]. Cai et al. proposed that miR-145 inhibits MAPK/ERK by targeting *IRS1* in PCOS [49]. Wang et al. found that miR-145 targets *Crkl* to promote GC proliferation, differentiation, and steroidogenesis via JNK/p38MAPK [50]. The MAPK pathway also interfaces with less-characterized miRNA-target pairs that may have granulosa cell-specific functions. The diagram depicts miR-146b-5p targeting Golgi glycoprotein 1 (*Glg1*), a protein involved in vesicular trafficking and secretion [51]. Additionally, miR-873-5p is shown to regulate heme oxygenase-1 (HO-1), an enzyme critical for cellular stress response and antioxidant defense [52]. While these interactions have been documented in cancer and endothelial cell lines, their roles in granulosa cell oxidative stress responses during follicular development remain unexplored. The presence of miR-483-5p in the pathway network without an explicit target suggests additional regulatory complexity that warrants further investigation. These emerging miRNA-target pairs highlight knowledge gaps that must be addressed to fully understand MAPK-mediated control of granulosa cell fate. The miRNAs regulating the MAPK signaling pathway are illustrated in Figure 3.

#### 2.2.4. TGF-β/SMAD Signaling Pathway

TGF-β/SMAD signaling is fundamental to female fertility. miR-543-3p targets decorin (*DCN*), leading to reduced expression of TGF-β pathway-related genes [53]. Du et al. reported that miR-130a promotes GC apoptosis by inhibiting TGF-β1 expression [54]. Wang et al. identified miR-2337 as a small activating RNA that enhances TGF-β1 promoter activity [55]. Several miRNAs target SMAD proteins: Nie et al. showed miR-23a and miR-27a promote apoptosis by targeting *SMAD5* [56]; Yu et al. found miR-30d-5p targets *SMAD2*, inhibiting proliferation and promoting apoptosis [57]; Yao et al. showed miR-181b-induced *SMAD7* downregulation controls GC apoptosis [58]; Liu et al. reported miR-92a inhibits apoptosis by targeting *SMAD7* [59]; Yin et al. demonstrated that miR-224 targets *SMAD4*, regulating GC proliferation and estradiol production [60].

**Figure 4:**
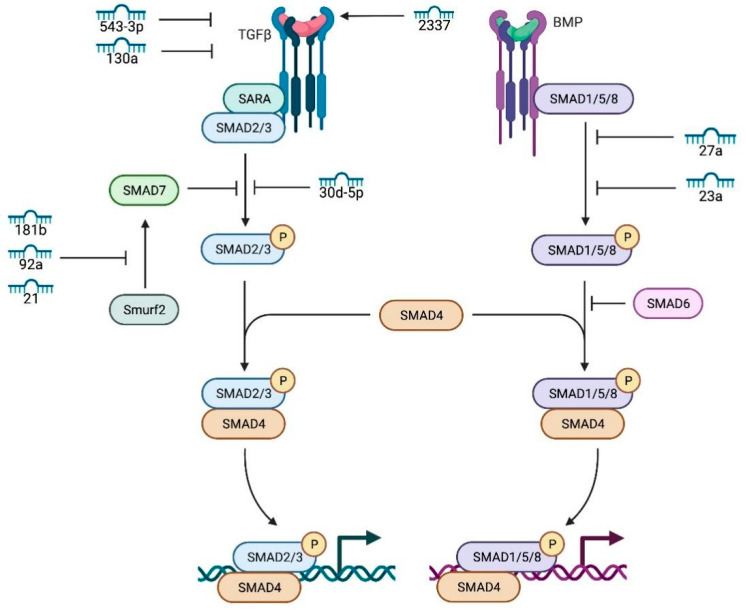
miRNAs regulating the TGF-β1 signaling pathway. This schematic depicts miRNA regulatory nodes within the canonical TGF-β/SMAD cascade. Ligand binding to the TGF-β/BMP receptor complex recruits the SARA adaptor protein, facilitating phosphorylation of SMAD1/5/8 (BMP pathway) and SMAD2/3 (TGF-β pathway). Phosphorylated R-SMADs form heteromeric complexes with SMAD4, translocating to the nucleus to regulate transcription. The inhibitory arm comprises SMAD6, SMAD7, and the E3 ubiquitin ligase Smurf2. Multiple miRNAs interface with this network: miR-130a targets SARA; miR-30d-5p and miR-23a target SMAD2/3; miR-27a, miR-181b, miR-92a, and miR-21 target SMAD7; 543-3p and 33 are shown as pathway-associated miRNAs without explicit target connections. The diagram uses numerical miRNA identifiers, phosphorylation is denoted by “P”, and connection directionality is not specified. Key takeaway: This network demonstrates multi-level miRNA control targeting adaptor proteins, signal-transducing SMADs, and inhibitory SMADs, thereby regulating signal flow through the TGF-β pathway.

TGF-β1 also trans-regulates miRNA expression: Du et al. found that TGF-β1 induces miR-764-3p expression, which enhances GC proliferation via SMAD4 while inhibiting estradiol synthesis via SF-1 [61]. Yin et al. showed miR-383 transactivates miR-320, inhibiting estradiol synthesis and proliferation by targeting *E2F1* and *SF-1* [62]. Importantly, *SMAD4* activates Wnt signaling to inhibit GC apoptosis [61] and represses multiple miRNAs that negatively regulate TGF-β signaling [63]. Recent evidence identifies the TGF-β1–SMAD4–BMF module as a conserved pro-survival axis in ruminant and human GCs [64,65,66]. The TGF-β/SMAD network also includes miRNAs with less-defined roles in granulosa cells. Figure 4 places miR-33 within the pathway without specifying a direct molecular target, suggesting it may function as a fine-tuner of overall pathway activity rather than a single-gene regulator. Studies in ovarian granulosa cells indicate that miR-33 can modulate TGF-β signaling by targeting key components of the pathway. For instance, in the context of polycystic ovary syndrome, miR-33b is upregulated and directly targets the TGF-β receptor *TGFBR1*, thereby influencing the signaling cascade and cellular outcomes [67]. In the ovarian context, miR-33 expression has been detected in follicular fluid exosomes, though its specific granulosa cell targets remain uncharacterized. Elucidating the mechanism by which miR-33 interfaces with the TGF-β/SMAD cascade could reveal novel regulatory nodes controlling follicular selection and atresia.

### 2.3. MiRNAs Regulating Steroidogenesis and Oxidative Stress Homeostasis

#### 2.3.1. Steroid Hormone Production

MiRNAs critically regulate estrogen synthesis by targeting *CYP19A1* (aromatase) and *CYP11A1*. Several miRNAs directly target *CYP19A1*: miR-210, miR-32, miR-146b, miR-10b, and miR-378 reduce estradiol secretion and promote apoptosis [68,69,70]. Others target transcriptional regulators: miR-29a impairs *CREB1* to inhibit *CYP19A1* [71]; Ding et al. showed miR-202-5p transactivated by *SF-1* targets *TGFβR2* [72]; Zhang et al. found miR-17-5p targets *E2F1* [73]; Liu et al. reported miR-1275 impairs *LRH-1*/*CYP19A1* axis [74]; Wang et al. showed miR-27a-3p targets *CREB1* [75]; and Du et al. found miR-764-3p targets *SF-1* [76]. Circular RNAs also participate: Zhang et al. identified hsa_circ_0043532 upregulating *CYP19A1* by acting as a ceRNA for miR-1270 [77], while Tao et al. revealed the KRTAP5-AS1/miR-199b-5p/*CYP19A1* axis [78]. Conversely, some miRNAs promote steroidogenesis: Zhang et al. showed miR-320a targets *RUNX2* to enhance steroidogenesis [79]; Wu et al. found miR-132 targets *Nurr1* [80]; and Dai et al. demonstrated miR-133b targets *Foxl2* [81]. A 2024 study in chickens showed miR-7 promotes apoptosis and autophagy by targeting *KLF4* via JAK/STAT3 [82].

#### 2.3.2. Reactive Oxygen Species (ROS) Balance

MiRNAs regulate ROS metabolism, critical for GC survival. Liu et al. reported that miR-27 increases ROS stress and apoptosis by targeting *SPRY2* and downregulating the p-ERK/Nrf2 pathway [83]. Ding et al. found hAMSC exosome-derived miR-320a targeting SIRT4 reduces ROS production [84], while hUMMSC exosome-derived miR-17-5p targeting *SIRT7* exerts similar effects [85]. *FOXO1* is a key mediator: it induces *TXNIP* expression, activates *NLRP3* inflammasomes, causing oxidative stress and apoptosis [86]. Several miRNAs target *FOXO1*: Yan et al. found miR-1224-5p reduces PCOS by inhibiting *NLRP3* activation via *FOXO1* [87]; Wei et al. showed miR-221-3p reduces ovarian reserve by targeting *FOXO1* in elderly women [88]; and Zhang et al. found miR-181a increases *FOXO1* acetylation and promotes apoptosis through *SIRT1* downregulation [77]. A 2025 study demonstrated that miR-361-5p alleviates GC dysfunction in diminished ovarian reserve by targeting *SLC25A24* to maintain mitochondrial function [89].

## 3. Conclusions and Future Perspectives

This review establishes miRNAs as a decisive regulatory network governing GC proliferation, apoptosis, steroidogenesis, and oxidative stress responses. Integrated analyses identify miR-129, miR-30a-5p, and miR-146a-5p as master regulators in human GCs [6], while the TGF-β1–SMAD4–BMF module represents a conserved pro-survival axis across species [64,65,66]. MiRNAs target core components of PI3K/AKT/mTOR, TGF-β/SMAD, JAK/STAT, MAPK, and Hippo pathways, as well as key functional genes including *TP53*, *BCL2* family members, *KCNA5*, *Mfn1*/*2*, and steroidogenic enzymes *CYP19A1*/*CYP11A1*.

However, significant translational challenges remain. First, miRNA pleiotropy poses off-target risks—miR-21 alone has four validated targets (*PTEN*, *SMAD7*) in PCOS [90,91]. Second, the vast majority of evidence derives from in vitro cultures or animal models, creating a substantial translational gap [1]. Third, the absence of follicle-specific delivery systems limits therapeutic applicability. Recent advances in tissue-specific miRNA delivery using cationic polymer nanocomplexes and peptide-modified AAV variants offer promise, but require further optimization for ovarian applications [3].

Future research must prioritize validation in human-relevant models. The development of human “ovaroid” organoids from induced pluripotent stem cells without exogenous transcription factors represents a crucial step toward bridging this gap [92]. Single-cell and spatial transcriptomics will further refine our understanding of cell type-specific miRNA functions within the follicular niche [2,6]. Addressing these challenges is paramount before miRNA-based strategies can be translated into viable clinical tools for managing PCOS, POF, and other ovarian disorders.

## Data Availability

No new data were created or analyzed in this study.

## Abbreviations

The following abbreviations are used in this manuscript:

miRNAs	MicroRNAs
GCs	Granulosa Cells
CGCs	Cumulus Granulosa Cells
mGCs	Mural Granulosa Cells
POF	Premature Ovarian Failure
PCOS	Polycystic Ovary Syndrome
POI	Premature Ovarian Insufficiency
FSH	Follicle-Stimulating Hormone
LH	Luteinizing Hormone
GnRH	Gonadotropin-Releasing Hormone
E2	Estradiol
P4	Progesterone
IGF-1	Insulin-like Growth Factor 1
IGF-1R	Insulin-like Growth Factor 1 Receptor
TP53	Tumor Protein P53
BCL2	B-cell Lymphoma 2
BAX	BCL2-Associated X Protein
KCNA5	Potassium Voltage-Gated Channel Subfamily A Member 5
Mfn1/2	Mitofusin 1/2
CYP19A1	Cytochrome P450 Family 19 Subfamily A Member 1 (Aromatase)
CYP11A1	Cytochrome P450 Family 11 Subfamily A Member 1
SF-1	Steroidogenic Factor-1 (NR5A1)
FOXO1	Forkhead Box O1
SIRTs	Sirtuins
Nrf2	Nuclear Factor Erythroid 2–Related Factor 2
PI3K/AKT/mTOR	Phosphoinositide 3-Kinase / Protein Kinase B / Mammalian Target of Rapamycin
TGF-β/SMAD	Transforming Growth Factor Beta / Mothers Against Decapentaplegic Homolog
JAK/STAT	Janus Kinase / Signal Transducer and Activator of Transcription
MAPK	Mitogen-Activated Protein Kinase
ERK	Extracellular Signal-Regulated Kinase
Abbreviation	Full Term
ROS	Reactive Oxygen Species
mt-OXPHOS	Mitochondrial Oxidative Phosphorylation
ATP	Adenosine Triphosphate
cAMP	Cyclic Adenosine Monophosphate
ECM	Extracellular Matrix
HPO axis	Hypothalamic-Pituitary-Ovarian axis
KGN cells	Human Granulosa-like Tumor Cell Line
BMSC	Bone Mesenchymal Stem Cell
ART	Assisted Reproductive Technology
scRNA-seq	Single-Cell RNA Sequencing
